# Signalling C-Type Lectins in Antimicrobial Immunity

**DOI:** 10.1371/journal.ppat.1003417

**Published:** 2013-07-25

**Authors:** Rebecca A. Drummond, Gordon D. Brown

**Affiliations:** University of Aberdeen, Aberdeen Fungal Group, Section of Immunity and Infection, Institute of Medical Sciences, Aberdeen, Scotland; The University of North Carolina at Chapel Hill, United States of America

## Introduction

Since it was first proposed that the innate immune system could recognise conserved microbial-associated molecular patterns (or PAMPs) through inherited receptors expressed by the host (termed pattern recognition receptors, or PRRs), several families of PRRs have been discovered and characterised. The most famous of these are the Toll-like receptors (TLRs), but there is growing appreciation that another large family of PRRs, known as the C-type lectin receptors (CLRs), also play a major role in antimicrobial immunity. CLRs have one or more carbohydrate recognition domains (CRDs) that recognise a wide variety of carbohydrate ligands. Other members of the CLR family, which do not recognise carbohydrate ligands but contain similar protein folds called C-type lectin-like domains (CTLD), have also been discovered and are included in this large family whose members are divided into 17 groups relating to phylogeny and structure. Upon ligand binding, some CLRs (such as Dectin-1, Dectin-2, and Mincle) undergo intracellular signalling to drive cellular responses. Here, we outline the signalling pathways downstream of these receptors and discuss how they, and some other CLRs (including the Mannose Receptor, CLEC5A, CLEC9A, and DC-SIGN), contribute to immunity against fungi, bacteria, viruses, and parasites.

## CLRs Can Initiate Complex Intracellular Signalling Pathways

Signalling by CLRs has multiple cellular consequences, including phagocytosis, activation of innate killing mechanisms (e.g., the respiratory burst), and inflammatory mediator production. Although incompletely understood, CLRs can trigger intracellular signalling through integral signalling motifs (such as immunoreceptor tyrosine-based activation motifs, or ITAMs) or by association with signalling adaptor molecules (such as the ITAM-containing FcRγ) [1], [2].

One of the best characterised signalling pathways downstream of CLRs is the Syk kinase/CARD9 pathway that is utilised by several CLRs including Dectin-1 (gene symbol *Clec7a*), Dectin-2 (*Clec4n*), and Mincle (*Clec4e*) [1], [2]. Syk is recruited following phosphorylation of tyrosines in the ITAM motif of these receptors, and the subsequent activation leads to the formation of a trimolecular complex composed of CARD9, Bcl10, and MALT1. This complex in turn is involved in the activation of NFκB, a major inflammatory transcription factor driving production of IL-6, pro-IL-1β, and TNFα (among others) [2].

Of particular recent interest is the ability of CLRs, such as Dectin-1, to activate inflammasomes—intracellular proteolytic complexes that utilise caspase-1 to generate active IL-1β. However, the process of inflammasome activation can be complex, as Dectin-1-dependent activation of the NLRP3 inflammasome was shown to require phagocytosis, suggesting that additional intracellular mechanism(s) were involved in these responses. On the other hand, Dectin-1 was also recently shown to activate a novel inflammasome involving caspase-8—a response that did not require internalisation, suggesting a direct extracellular sensing mechanism [3].

The importance of these pathways has been highlighted by the phenotypes observed in murine gene knockout models and with human mutations. For example, a deficiency in CARD9 significantly enhances susceptibility to fungal infections in both mice and humans, and is more severe than a deficiency in any single receptor, indicating that redundancy between CLRs is common [2], [4] and also highlighting the possible involvement of other non-CLR receptors that utilise CARD9. Indeed, pathogens possess multiple PAMPs and will engage several PRRs during an immune response, and this allows for “fine-tuning” of signalling that shapes the cellular response appropriately. CLR cross-talk can be critical to protection, and several examples of this have recently been described [5]. The medical relevance of CLR signalling means that further understanding of these processes is essential if we are to gain insights into how stimulating different CLRs gives varying responses despite the utilisation of common signalling pathways.

## Fungi

CLRs have been best studied in the context of fungal infections—an area of research which has gained increasing attention due to the substantial increase in the number of life-threatening fungal infections in recent years. Several CLRs have been implicated in antifungal immunity and include Dectin-1, Dectin-2, the Mannose Receptor (MR), and Mincle (Table 1). Dectin-1 is the most extensively characterised antifungal receptor and has been shown to play vital roles in the defence against an array of fungal pathogens, including *C. albicans*, *A. fumigatus*, and *P. carinii* [6]. Dectin-1 recognises fungi by binding β-glucans in the fungal cell wall, and subsequently signals through Syk/CARD9 to induce cellular responses including phagocytosis, induction of the respiratory burst, and cytokine production. Another recently described function is the ability of Dectin-1 to directly induce adaptive Th1 and Th17 responses [2]. Dectin-1^−/−^ mice have been used to demonstrate how these functions are important for antifungal immunity *in vivo*. Inefficient fungal uptake and killing in Dectin-1^−/−^ mice leads to uncontrolled fungal growth in mouse models of systemic candidiasis and aspergillosis, leading to an enhanced rate of mortality [6]. Furthermore, Dectin-1^−/−^ mice and humans with a mutation in the Dectin-1 gene (leading to a non-expressed truncated form of Dectin-1) can present with an increased susceptibility to mucosal candidiasis and aspergillosis [2], [6], in part due to aberrant induction of adaptive immunity.

**Table 1:** Selected CLRs, their microbial ligands, and the microbes that have altered ability to infect in the absence of the receptor within mice and humans.

CLR	Identified Microbial Ligand(s)	Infections with Altered Susceptibility in the Mouse KO	Infections Linked to Human Polymorphisms	Selected References
Dectin-1	β-glucan (fungi)	↑ *C. albicans*↑ *A. fumigatus*↑ *P. carinii*	↑ *C. albicans*↑ *A. fumigatus*	[6]
Dectin-2	α-mannan	↑ *C. albicans*	None identified/confirmed	[7]
DC-SIGN*	ManLAM (mycobacteria)gp120 (HIV)	↑ *Mycobacterium tuberculosis*	↓ *M. tuberculosis*↑ *HIV*	[10], [12], [24]
Mannose Receptor (MR)	Mannan/mannose	↑ *Cryptococcus neoformans*	↑ *M. tuberculosis*	[9], [11], [24]
CLEC5A	Dengue virus**	↓ Dengue virus***	None identified/confirmed	[15], [16]
CLEC9A	Unknown	↑ Vaccinia virus	None identified/confirmed	[17]
Mincle	α-mannose (fungi)TDM (mycobacteria)	↑ *C. albicans*↑ Mycobacterial antigens	None identified/confirmed	[1], [10]

A selection of CLRs are listed with identified microbial ligands and phenotypes observed with different pathogens in both mice and humans, where ↑ denotes enhanced susceptibility of the host and ↓ denotes enhanced resistance.

* DC-SIGN is a human molecule with related homologues in the mouse; mouse phenotypes described are for SIGNR3 knockouts.

** Exact ligand on DV is unknown.

*** These experiments were performed using blocking antibodies to CLEC5A. Abbreviations: HIV, human immunodeficiency virus; ManLAM, mannosylated lipoarabinomannan; TDM, trehalose-6, 6′-dimycolate.

Like Dectin-1, Dectin-2 also plays a key role in the protection against *C. albicans*, through innate recognition and the promotion of Th17 responses [7], [8]. The roles of the MR and Mincle are less well defined however. MR contains no known signalling motifs and it is unclear whether signalling occurs directly from the MR, and MR^−/−^ mice do not have increased susceptibility to most fungal pathogens, with the exception of *C. neoformans* [9]. Mincle, on the other hand, has contradicting studies on the species it recognises [10] possibly because of fungal strain variation, which can be important to the interpretation of these types of studies [6]. Defining the roles of these receptors will hopefully help feed development of novel therapeutics and vaccines, of which there are currently few effective options for the former, and none for the latter.

## Bacteria

The majority of studies analysing the role of CLRs in bacterial infections have focused on mycobacterial diseases, and we will focus on this data here. However it is important to note that, although less well characterised, CLRs have been implicated in the recognition of other bacterial species. For example, the MR recognises a number of bacteria including *Klebsiella* and *Streptococcus* species [11].

Several CLRs recognise mycobacteria and play roles in the resulting immune responses. The MR has been shown to mediate the binding and internalisation of mycobacteria, and plays a role in antigen presentation and the promotion of T-cell responses [11]. Mincle and Dectin-1 have also been linked to mycobacterial recognition, although Mincle's ligand is the only one to be identified (mycobacterial cord factor, also known as TDM). Despite their ability to recognise mycobacteria, none of the receptor-deficient mouse models exhibit increased susceptibility to infection [10], [12]. This suggests that other receptors are able to compensate, and that there is a high level of redundancy for mycobacterial recognition. However, while individual receptors appear redundant, the shared downstream signalling pathway appears to be central for protection as CARD9^−/−^ mice are highly susceptible to mycobacterial infection [10].

## Viruses

Unlike fungi and bacteria in which these receptors are thought to play protective roles, many CLRs involved in viral recognition appear to promote transmission and infection. One of the best characterised examples is DC-SIGN and HIV. Viral particles bind DC-SIGN via gp120 on the viral surface before being endocytosed by dendritic cells (DCs), and HIV is then thought to use the DC as a safe mode of transport to lymph nodes where it comes into contact with its target CD4^+^ T-cells. Indeed, various human DC-SIGN polymorphisms have been associated with increased transmission and binding of HIV [13]. Other examples include binding of dengue virus (DV) by CLEC5A and the MR, and blocking these CLRs in experimental models prevented viral growth and associated lethal inflammation [14]–[16].

On the other hand, CLRs can also play protective roles during viral infections, some through indirect mechanisms. For example, CLEC9A (also known as DNGR-1) was recently shown to facilitate antigen cross-presentation, a function required for the control of infections with vaccinia virus [17] and herpes simplex virus [18].

## Parasites

Many parasites express a large number of carbohydrates, and therefore have the potential to engage host CLRs. Dectin-2, while typically considered an antifungal CLR, can also contribute to immunity against parasitic worms. Dectin-2 was recently shown to recognise a component of *Schistosoma mansoni* egg antigen (SEA), and was responsible for the subsequent activation of the NLRP3 inflammasome. While absence of this inflammatory response did not alter parasite burden, it did alleviate Th2-mediated immunopathology [19]. Interestingly, Dectin-2 has also been shown to promote Th2 responses to the common allergen house dust mite (HDM) antigen [2], while Th17 responses are seen with fungi (see above). However, it is not well understood how Dectin-2 can promote opposing T-cell responses to different antigens, although further definition of the downstream signalling pathways following recognition should help answer these questions.

Like Dectin-2, other CLRs have also been found to modulate adaptive immune responses to parasites. For example, the MR was shown to help drive the protective Th2 response during *S. mansoni* infection and enhance uptake of infectious cercariae [20]. CLEC9A, on the other hand, was shown to drive CD8^+^ T-cell activation (possibly through previously described cross-presentation functions) during cerebral malaria in mice, resulting in immunopathology and mortality [21].

## Concluding Remarks

CLRs are involved in immunity to several classes of microbe, from initial recognition and uptake to modulation of adaptive immunity. Indeed, although not discussed here, CLRs have also been implicated in maintaining immune homeostasis through recognition of endogenous ligands; both Mincle and CLEC9A are involved in the recognition of necrotic cells by binding exposed nuclear proteins and actin filaments, respectively [22], [23]. However, much is still to be learned about these innate receptors, such as the specific outcomes of cross-talk during signalling and how this is regulated. Furthermore, microbial ligands for many CLRs remain unidentified. Unveiling the microbial components that stimulate potent immune responses via CLRs is an important avenue for research into novel vaccines and therapies. Moreover, as our knowledge grows it is likely that we will identify additional human CLR polymorphisms that cause defects in antimicrobial immunity.
